# Extracellular Signal-Regulated Kinase: A Regulator of Cell Growth, Inflammation, Chondrocyte and Bone Cell Receptor-Mediated Gene Expression

**DOI:** 10.3390/ijms20153792

**Published:** 2019-08-03

**Authors:** Nathan Lu, Charles J. Malemud

**Affiliations:** Department of Medicine, Division of Rheumatic Diseases, Case Western Reserve University School of Medicine, University Hospitals Cleveland Medical Center, Cleveland, OH 44106, USA

**Keywords:** arthritis, bone, cancer, chondrocyte, ERK1/2, gene expression, inflammation

## Abstract

Extracellular signal-regulated kinase (ERK) is a member of the mitogen-activated protein kinase family of signaling molecules. ERK is predominantly found in two forms, ERK1 (p44) and ERK2 (p42), respectively. There are also several atypical forms of ERK, including ERK3, ERK4, ERK5 and ERK7. The ERK1/2 signaling pathway has been implicated in many and diverse cellular events, including proliferation, growth, differentiation, cell migration, cell survival, metabolism and transcription. ERK1/2 is activated (i.e., phosphorylated) in the cytosol and subsequently translocated to the nucleus, where it activates transcription factors including, but not limited to, ETS, c-Jun, and Fos. It is not surprising that the ERK1/2 signaling cascade has been implicated in many pathological conditions, namely, cancer, arthritis, chronic inflammation, and osteoporosis. This narrative review examines many of the cellular events in which the ERK1/2 signaling cascade plays a critical role. It is anticipated that agents designed to inhibit ERK1/2 activation or p-ERK1/2 activity will be developed for the treatment of those diseases characterized by dysregulated gene expression through ERK1/2 activation.

## 1. An Overview of ERK Signaling Components

Extracellular signal-regulated kinase 1/2 (ERK1/2) is a member of the “generic” mitogen-activated protein kinase (MAPK) signaling pathway [1]. The MAPK signaling pathway also includes among its members p38, MAPK, and c-Jun-N-terminal kinase 1/2/3 (JNK1/2/3). Although their sequence similarity overlaps with ERK1/2, ERK-3, -4, -7 and -8 are not considered “genuine” members of the MAPK kinases. In contrast, ERK-5 does belong to the MAPK group. Thus, the atypical forms of ERK are ERK-3, -4, [2] as well as ERK-7 [3] and NF-κB essential modulator (Nemo)-kinase-1 [4], defined as atypical on the basis of being phosphorylated by members of the upstream MAP kinase kinases, (MAPKK/MEK) [5], as is ERK1/2, whereas ERK-5 was shown to be activated by MEK5 [5].

MAPK signaling regulates many cellular events such as cell proliferation, differentiation, cell migration, controlled cell death (apoptosis), and senescence [6], as well as certain aspects of cell cycle progression, cell survival, metabolism and transcription [6]. The sizes of ERK1 and ERK2 were originally identified by 1-Dimensional and 2-Dimensional gel electrophoresis as migrating between 41 and 45 kilodaltons, as well as by comparing the migration rates of ERK1 (p44^mapk^) and ERK2 (p42^mapk^), respectively, on SDS-PAGE, when the migration rates of ERK1 and ERK2 were compared alongside molecular weight standards.

**Figure 1:**
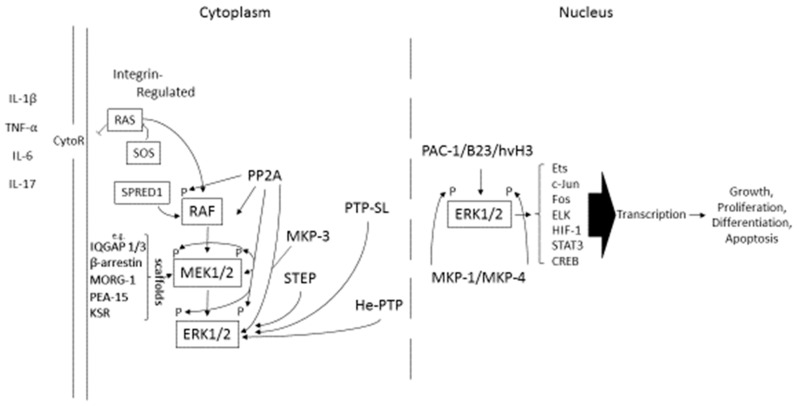
Activation and Regulation of the ERK1/2 Signaling Cascade. Pro-inflammatory cytokines are elevated in various states of chronic inflammation. These cytokines are exemplified by IL-1β [20], TNF-α [20], IL-6 [21], and IL-17 [22]. They interact with their respective receptors (CytoR) on the plasma membrane of many cell types to initiate the ERK signaling cascade. Figure 1 was adapted from a figure produced by ABCAM, Inc. (www.abcam.com). Permission to publish this schematic was solicited and approved by ABCAM.com.

ERK1/2 proteins are serine/threonine protein kinases that are a component of the rapidly accelerated rat sarcoma (RAS)-rapidly accelerated fibrosarcoma (RAF)-Mitogen-mitogen protein kinase (MEK1/2)-ERK1/2 signaling cascade [7]. Activation of the RAS-RAF-MEK1/2-ERK1/2 signaling cascade can occur in response to pro-inflammatory cytokine/cytokine receptor-mediated activation (Figure 1). In this schematic model of the ERK1/2 signaling cascade, MEK1/2 resides upstream of ERK1/2, wherein MEK1/2 catalyzes the phosphorylation of human ERK1/2 at Tyr^204/187^ and then at Thr^202/185^ [1,7]. Conversely, the regulation and activation of the atypical ERKs, ERK3 and ERK4, was attributed to the activity of MAP kinase-activated protein kinase 5 (MK5) [5].

The ERK1/2 signaling cascade occurs through a mechanism involving phosphorylation at both of the Tyr/Thr sites. This mechanism was found to be required for full activation of ERK1/2 [7]. This primary activation step also requires efficient D-site and F-site docking. Thus, ERK1/2 have a D-site recruitment site that binds to the D-site docking domain of certain substrates as well as an F-site recruitment site that binds to the F-site docking domain of these substrates. This provides a mechanism for the engagement and facilitation of efficient site-specific phosphorylation at specific serine/threonine-proline sequences by their cognate kinases, which exists in contrast to other MAP kinase substrates [6].

Several additional proteins play important roles in downstream ERK1/2 signaling. These include scaffold proteins exemplified in Figure 1 by Kinase Suppressor of RAS1/2 (KSR1/2). KSR1/2 interacts with RAF-1, MEK1/2, ERK1/2 and RAS-GTPase-activating-like protein (IQGAP3), a modulated protein associated with ERK1/2 signaling that acts to maintain activated ERK1/2 in the cytoplasm. In addition, ß-Arrestin1/2, Modular Construction of a Signaling Scaffold (MORG-1), and Phosphoprotein Enriched in Astrocytes-15 (PEA-15) have all been implicated as regulators/modifiers of activated ERK1/2 [7] (Figure 1). In addition to those shown here (Figure 1) as representative of MEK1/2/ERK1/2 scaffold proteins, several other scaffold proteins have been identified [8,9], including IQGAP2 [9], Shoc2/SUR8 [10], MP1/p14 [11], caveolin-1 [12], PRO40 [13], prohibitin (PHB) [14], and ASPP2 [15]. On the other hand, MAPK phosphatases are germane to the regulation of ERK1/2 and RAS. In this regard, MAPK phosphatase-3 (MKP-3) mediates a negative feedback loop involving the dephosphorylation of ERK1/2, whereas PP5 acts on RAF-1 by dephosphorylating Ser^338^, an essential activation site on RAF-1 [16]. For example, PEA-15 modifies ERK signaling by keeping ERK1/2 in the cytoplasm without blocking ERK activation, a finding that was originally reported by Formstecher et al. [17]. Furthermore, there are other cytoplasmic proteins, including the tyrosine phosphatases protein phosphatase-2 (PP2A) [18], Striatal-enriched protein tyrosine phosphatase (STEP), Phosphotyrosine-specific phosphatase (PIP-SL), and Haematopoietic Protein Tyrosine Phosphatase (HE-PTP), that differentially interact with phosphorylated ERK1/2 in the cytoplasm (Figure 1). Conversely, Sprouty-related EVH1 domain-containing protein 1 (SPRED1) binds to RAF-1 protein and not to ERK1/2, whereas Son of Sevenless (SOS) binds to RAS-GTPase [19], catalyzing the nucleotide exchange on RAS, forcing the release of the bound nucleotide (Figure 1). Several factors localized to the nucleus include MAP kinase phosphatase-1, -2 (MKP-1/MKP-2) and Pro-caspase-3 Activator (PAC-1). These phosphatases and others, including dual specificity phosphatases-B23/hVH3, modulate the activity of the phosphorylated ERK1/2, thus regulating ERK1/2 activity in the nucleus (Figure 1).

The translocation of phosphorylated ERK1/2 from cytosol to nucleus involves a distinct mechanism whereby a nuclear translocation signal interacting with importin-7 provides the mechanism for stimulated translocation through nuclear pores [23,24,25,26]. In the case where this mechanism does not work properly, cytosolic retention of ERK1/2 means that certain nuclear substrates are not activated [15], leaving cytosolic phosphorylated ERK1/2 capable of “potentiating the catalytic activity of certain proapoptotic proteins including cytosolic Death-associated protein kinase 1” [25]. ERK1/2 translocation to the mitochondria and Golgi has also been reported [26,27]. Zuckerbraun et al. [27] reported that the small GTPase RhoA, a regulator of actin cytoskeletal activity, may be responsible for the subcellular deposition of ERK and its effect on smooth muscle cell proliferation. Of note, the translocation of ERK5 is regulated by a different nuclear localizing and nuclear export mechanism involving importin-α/β, whereas ERK1/2 uses importin 7 [28].

Phosphorylated ERK1/2 has the capacity to activate several transcription factors [29], including E26 transformation-specific or E-twenty-six (Ets-1), Jun-proteooncogene (c-Jun), Fos-proteooncogene (Fos), E-26-oncogene (Elk), Hypoxia-inducible-factor-1 (HIF-1), cyclic AMP receptor binding protein (CREB), and signal transducer and activator of transcription-3 (STAT3) (Figure 1).

Several levels of engagement regulate ERK1/2 activation (i.e., phosphorylation), as shown in Figure 1. For example, there are the RAS-integrin and the Son of sevenless (SOS) protein positive interplays [30]. In addition, RAS guanyl-releasing protein-1 (RAS; RAS-GRF1) acts on the membrane-bound GTPase RAS. In this way, RAS-GRF1 contributes to the activation of ERK1/2 [4]. In turn, phosphorylated RAS-1 regulates the activation of MEK1/2, whose activity is required for full activation of ERK1/2 (p-ERK1/2). Conversely, Sprouty-related proteins with EVH1 domain (Spred1) [31,32] acts as a negative regulator of the RAS-ERK pathway [32]. Several scaffolding proteins are shown in Figure 1, including RAS-GTPase-activating-like protein-1 -3 (IQGAP1/3), β-arrestin [33], and MORG-1 [34]. Kinase suppressor of RAS (KSR; KSR-1, KSR-2) [35,36] can interact with RAF-1, MEK1/2 and ERK1/2. Of note, IQGAP1 was shown to interact with ERK2, which did not require ERK2 to be phosphorylated [37], while IQGAP3 can specifically interact with the active GTP-bound form of RAS [38]. Furthermore, ERK acts downstream of IQGAP3 [38]. MORG-1 is also a facilitator of ERK1/2 activation in that MORG-1 specifically interacts with several up-stream regulators of ERK, such as RAF-1, MEK, and ERK, which stabilizes their formation into oligomeric complexes [35]. PEA-15 represents another facilitator of ERK1/2 activation [39] by the tethering of ERK1/2 to the cytoplasm, thus regulating p-ERK1/2 translocation to the nucleus. Serine/threonine protein phosphatase 2A (PP2A), PTP-SL, Striatal-Enriched protein tyrosine phosphatase (STEP), and HePTP are protein tyrosine phosphatases that act on MAP kinase substrates. For example, STEP targets ERK1/2 causing it to be inactivated [40]. These phosphatases regulate ERK by binding to ERK through a kinase-interaction motif (KIM) located in the non-catalytic regulatory domain. For example, PTP-SL acts to retain ERK and other MAP kinases in the cytoplasm in an inactive form through their association with the KIM and through the dephosphorylation of tyrosine residues. Of note, PTP-SL and STEP are related to one another. They are phosphorylated by the cAMP-dependent protein kinase A. Additional regulators of ERK, exemplified by RAS-GAP belonging to the GAP1 family of GTPase-activating proteins, PP5, and hSef, which binds to activated forms of MEK1/2, inhibiting the dissociation of the ERK/MEK complex which prevents nuclear translocation of ERK [41], are also critical in regulating the functional contribution of ERK1/2 to gene transcription.

Following the detachment of activated ERK1/2 from proteins that anchor it to the cytoplasm, an additional phosphorylation of 2 serine residues in a domain known as the SPS motif residing within a nuclear translocation signal (NTS) of ERK1/2 occurs [13]. This added phosphorylation step provides the means for ERK1/2 to interact with importin7, which promotes nuclear translocation. Thus, the translocation of p-ERK1/2 to the nucleus is the mechanism whereby several transcription factors (TFs), shown in Figure 1 including Ets, c-Jun, Fos, ELK, HIF-1, STAT3, and CREB, are activated. TFs activate genes that control cell proliferation growth, apoptosis, and differentiation. Thus, providing STAT3 as a site for phosphorylation in the cytoplasm is another important function of activated ERK1/2 in the nucleus [42].

Once p-ERK1/2 is translocated to the nucleus, several phosphatases can further regulate the activity of p-ERK1/2 [43]. Thus, PAC-1, also called (DUSP2), is an inducible, nuclear-specific, dual-specificity MAPK phosphatase. B23 is a multifunctional nucleolar protein that is overexpressed in numerous cancers and is associated with tumorigenesis. Another inducible nuclear phosphatase is hVH3, also called DUSP5 [43]. The dephosphorylation of ERK1/2 is also mainly carried out by protein-tyrosine-specific phosphatases, protein-serine/threonine phosphatases and/or dual specificity phosphatases (DUSPs), in a manner consistent with regulating the activity of several of the MAPKs [7]. DUSPs are divided into six subgroups, namely, phosphatases of regenerating liver (PRL), cyclin dependent phosphatases (e.g., Cdc14), phosphatase and tensin homolog deleted on chromosome 10 (PTEN), myotubularins, mitogen-activated protein kinase phosphatases (MKPs), and the so-called atypical DUSPs [43,44,45].

In addition to the important role played by DUSPs in regulating overall ERK activity, activated ERK1/2 catalyzes the phosphorylation of numerous nuclear transcription factors, exemplified by ETS; ELK-1, a member of the ETS oncogene superfamily) [46,47]; c-Fos, a component of the activator protein-1 (AP-1) complex [48]; and Forkhead Box O (FOXO) [49], all of which arise through both active and passive nuclear translocation of phosphorylated ERK1/2 [1,50]. Of note, constitutively activated RAS-RAF-MEK-ERK was found to be present in about 30% of all human cancer tissues [51]. Thus, the RAS-RAF-MEK-ERK pathway has become a critical target for anti-tumor therapy to the extent that the inhibition of mutant B-RAF (Val^600^→Glu) was found to be therapeutically efficacious [1] (see below).

### 1.1. Regulation of the RAS-RAF-ERK Pathway

The RAF family kinases are oncoproteins that were originally discovered almost 4 decades ago. RAF acts as a signaling relay kinase downstream of RAS, although ongoing studies have emphasized that current RAF inhibitors induce, rather than suppress, ERK1/2 signaling by stimulating RAF dimerization [52]. Despite this unexpected finding, genetically engineered mouse models showed how RAF kinases were critical for understanding the role played by RAS-RAF-ERK in embryonic development, as a regulator of tissue homeostasis as well as being crucial for promoting disease pathogenesis [52,53,54,55,56]. In fact, the dependency of tumor growth on the RAF interactome [55], which arose from the analysis of RAF-interacting proteins, has emerged as a promising area for protein-directed cancer therapy [57].

Considerable interest has also arisen regarding the specific targeting of RAF family proteins, including A-RAF, B-RAF and C-RAF [58]. A few studies have investigated targeting A-RAF because of its role in inhibiting apoptosis through its capacity to bind to serine-threonine kinase-2 (MST2). In this regard, MST2 was shown to protect against oncogenic transformation by suppressing ERK1/2 activation. However, a good deal of recent drug development in this area over the past 2 years or so has centered on B-RAF [57], because of its acknowledged role in colorectal cancer [58], non-small cell lung cancer [59] and melanoma [60,61], among other diseases.

Several additional aspects of RAS-RAF-ERK signaling have been investigated for their role in cellular transformation [62]. These include, but are not limited to, the putative differential functions of ERK1 and ERK2 [63], and importantly the level of “cross-talk” between the activated ERK1/2 pathway and the PI3K/Akt/PTEN/mTOR pathway [64,65,66,67], where substrates protein kinase B, also known as AKT, calmodulin-dependent protein kinase II (CaMKII), protein kinase A (PKA), Protein kinase C (PKC), and casein kinase-2 (CK2) are relevant to both the ERK1/2 and PI3K/Akt/PTEN/mTOR signaling pathways [67].

### 1.2. Inhibition of the ERK1/2 Pathway

The ERK1/2 signaling pathway plays a crucial role in aberrant cell proliferation, tumor development and gene expression because of how ERK signaling impacts cell cycling, cell migration, cell invasion [68], apoptosis [7], autophagy [69,70], and metastasis [71]. Thus, blocking the constitutively activated ERK pathway using upstream MEK inhibitors was shown to sensitize tumor cells to apoptosis induced by cytotoxic cancer drugs [72]. In addition to MEK inhibition, several other molecules germane to the ERK signaling pathway, including RAS [73], B-RAF kinase [74], DUSPs [44,75], tropomyosin receptor kinase (TRK) [76], and FOXO [49], are also being pursued as anti-cancer targets. Importantly, the use of combinations of cytostatic MEK inhibitors with more conventional microtubule destabilizing agents or histone deacetylase (HDAC) inhibitors has shown promise for reducing the level of cytotoxic anti-cancer drugs required to induce remission [72].

### 1.3. Role of the ERK1/2 Pathway in Inflammation

ERK1/2 signaling has also been implicated in several diseases that are characterized by a chronic state of inflammation such as rheumatoid arthritis (RA), psoriatic arthritis (PsA) and the progressive form of osteoarthritis (OA). In this regard, RA, PsA and the clinically apparent inflammatory component of OA are characterized by defective cellular and humoral immunity, which causes a state of chronic inflammation to ensue [77]. Special attention has also been paid to those environmental conditions that involve the innate immunity component Toll-Like Receptor (TLR) and TLR activation, the induction of pro-inflammatory cytokine gene expression as well as other soluble mediators of inflammation. For example, more than a decade ago, Lang et al. [78] reported that mice deficient in DUSP1, DUSP2, or DUSP10 exhibited local and systemic inflammation. ERK activation has also been associated with alcoholic-induced inflammation and alcoholic liver disease and its association with an increase in nuclear factor κB (NF-κB) and activator protein-1 (AP-1) activity [79]. More recently, Xu et al. [80] identified tumor progression locus 2 (TPL2), also known as Col-1, as a MAPK kinase kinase (MAP3K). TLP2 was activated in response to various receptors associated with inflammation, including TNFαR, ILR, TLR, CD40, IL17R, and G-protein coupled receptor (GPCR). In this regard, TPL2 was shown to regulate MEK1/2 and ERK1/2 in association with inflammatory responses.

#### TPL2/Pro-Inflammatory Cytokines/Inflammation

IκB kinase activity is required for the activation of the NF-κB complex. In this regard, Gantke et al. [81] showed that tumor progression locus-2 (TPL2), a molecule previously identified as being critical in inflammation, is a MAP-3 kinase component relevant to the TLR and TNF receptor activation of ERK1/2 [82] because TPL2 facilitated the phosphorylation of MEK. Of note, new evidence relating to the capacity of the pro-inflammatory cytokine IL-6 to induce RAS/ERK/CCAAT-enhancer-binding proteins (C/EBP) and activate T-helper cells, regulatory T-cells and T-helper17 (Th17) cells has also come to the fore [83], since elevated levels of IL-6 were found to be associated with inflammation in diseases such as RA, multiple sclerosis, asthma, inflammation-associated cancer [83], and inflammatory bowel disease [42,84,85]. It is also worth noting that the interplay that occurs between the JAK/STAT and ERK pathways has also been implicated in inflammatory bowel diseases, such as Crohn’s disease [42].

## 2. Novel Insights into the Regulation of ERK Activation

Newly recognized insights into the role of MAPK/MAPK activated protein kinases (MKs) heterodimeric protein kinase signaling complexes have emerged. The results of these analyses provided the structural underpinnings for understanding the role played by MK linear motifs, the role of ERK-independent activation of RAS-induced senescence via ribosomal S6 kinase (RSK), the role of RSK inhibitors in dampening ERK activity, how ERK via RSK can regulate cell motility [86], and how MK5/p38-regulated-activated protein kinase (PRAK) can act as a tumor suppressor [87].

KSR-1 and KSR-2 are also important regulators of RAS-mediated signaling of RAF/MEK/ERK. The regulation of the RAF/MEK/ERK pathway via KSR-1, -2 has been particularly germane for understanding the regulation and alteration of energy balance, with specific implications for obesity as well as cancer pathogenesis and progression [88]. Also, there has been a recent uptick in the potential regulation of ERK activity by KSR-1, -2, through the discovery of, and exploitation of, RAS-MAPK “oncomiRNAs” (oncomiRs), which may eventually be useful as an epigenetic regulator of oncogenic RAS-mediated signaling [89]. Of note, the discovery of the RAS-kinase inhibitory protein (RKIP) acting as an integrator of RAF-MEK-ERK signaling with the NF-κB signaling pathway [89,90] may eventually be useful for suppressing oncogenic transformation, metastasis and chronic inflammatory states.

## 3. Upstream Inhibitors of ERK

It was not unexpected that, with ERK signaling implicated in numerous cellular events related to aberrant cell behavior, ERK activation became a target for drug development. Thus, the development of upstream MEK inhibitors has become most prominent in this endeavor [91,92,93]. Although substantial progress was achieved using the MEK approach [94,95,96], additional, more compelling issues have arisen. For example, using different cell types and an ERK1/2 knockdown experimental design brought about by RNA interference, Hong et al. [97] found that ERK1/2 regulated MEK1 levels, which was partially a result of increased transcription. However, MEK2 downregulation was seen only at the protein level, suggesting a post-translational mechanism. Furthermore, MG132 and bortezomib, agents which block proteasome activity, produced a similar post-translational effect on MEK2.

## 4. Role of ERK Signaling as a Regulator of Chondrocyte Receptor-Mediated Gene Expression

ERK signaling is a critical component in the dysregulation of chondrocyte gene expression in states of chronic inflammation. This is clearly exemplified by the capacity of pro-inflammatory cytokines such as tumor necrosis factor-α (TNF-α), interleukin-1 (IL-1) and IL-6 to activate MAPKs, including ERK1/2 [98] (Figure 1). In one example, abnormal signaling by the Wingless-integrated (Wnt) family of proteins, most prominently by Wnt5a, in response to fibronectin fragments generated by enzymes resulted in the activation of both ERK and JNK [99]. Importantly, Wnt5a reduced the synthesis of the large sulfated-proteoglycan aggrecan, by suppressing transcription of the aggrecan (*ACAN*) gene whose expression is essential for maintaining the integrity of the extracellular matrix in articular cartilage [77]. Wnt5a has also been reported to stimulate matrix metalloproteinase (MMP)-1, -3, and -13 production, so that inhibition of ERK as well as JNK and p38, among the MAPKs, suppressed Wnt5a-induced MMP-1 and MMP-13 in normal human chondrocytes [100,101]. Thus, modulation of the MAPK cascade, including ERK, has been proposed as a mechanism required to blunt cartilage destruction in a variety of various pathological states, including inflammatory arthritis [101,102,103,104].

### 4.1. Regulation of MMPs and A Disintegrin and Metalloproteinase with Thrombospondin Motif (ADAMTS)

A slew of experimental evidence now implicates MMPs and another family of enzymes named A Disintegrin and Metalloproteinase with Thrombospondin Motif (ADAMTS) in various forms of inflammatory arthritis [101,102,103,104]. There is also persuasive evidence from both in vitro and in vivo studies linking MMP gene expression to the skewing of chondrocyte and osteoblast homeostasis towards a catabolic state [101] that is regulated, in part, by activation of the MAPK cascade, with ERK1/2 playing a crucial role [105]. Thus, Prasadam et al. [104] showed that up-regulation of ADAMTS and MMPs correlated with activation of ERK1/2 signaling, which could be inhibited by the ERK small molecule inhibitor PD98059 in subchondral bone osteoblasts. In fact, ERK inhibition altered the production of ADAMTS and MMPs in OA cartilage as well. The results of another study showed that high molecular weight hyaluronan inhibited IL-6-IL-6R-induced MMP production in human chondrocytes by increasing the activity of the ERK inhibitor MKP-1 [105].

### 4.2. Epigenetic Regulation of ERK1/2

Epigenetics was also found to play a role in the regulation of MMPs via ERK1/2. Thus, depletion of chondrocyte HDAC3 resulted in the hyperphosphorylation of ERK1/2 and its downstream substrate Runt-related transcription factor 2 (Runx2) [106], an effect which could be mimicked by the ERK1/2-specific phosphatase, DUSP6 [107]. In addition, ERK1/2 inhibitors and DUSP6-adenovirus-transfected cells reduced *MMP-13* gene expression. Wang et al. [108] confirmed and extended the results of the abovementioned study [107] by showing that HDAC4 and HDAC8 were also upstream regulators of ERK activity that mediated IL-1β-IL-1β receptor-stimulated *ADAMTS-4, -5* gene expression. Of note, it had been previously shown that HDAC inhibitors suppressed uniaxial cyclic tensile strain-induced p38, ERK, and c-Jun amino terminal kinase (JNK) in human chondrocytes [109]. In fact, Ma et al. [110] had previously shown that hydrostatic pressure significantly increased the activation of ERK1/2 without altering ERK content in cultured condylar chondrocytes. Hydrostatic pressure also resulted in the activation of focal adhesion kinase (FAK) and PI3K, as well as the mechanosensitive molecules integrin α2, integrin α5, and integrin β1. Impact loading of chondrocytes also caused a 5.8-fold increase in ERK-1 and a 5.4-fold increase in a nearby zone of whole cartilage slices in vitro [100], whereas impact loading led to a 4-fold increase in ERK2 and a 3.6-fold increase in a zone of cartilage farther away from the zone of impact. The increase in ERK1 and ERK2 activation was accompanied by an increase in the expression of *MMP-13, ADAMTS-5* and *TNF-α* genes. Thus, the significance of abnormal loading of joints via increasing hydrostatic pressure or sustained impact loading has long been proposed as a potential initiator of extracellular matrix damage and controlled chondrocyte death in articular cartilage, which would precede alterations in joint cartilage consistent with the development of OA [110,111,112].

Taken together, these results could be interpreted as meaning that abnormal mechanical signals cause damage to articular cartilage that involves ERK activation in cartilage subsequent to trauma. In yet another study, vorinostat, an inhibitor of HDAC, selectively inhibited IL-1β-induced ERK1/2 and p38 MAPK activation, but not JNK activation in human OA chondrocytes [113]. These results suggested yet another mechanism involving HDAC that could be targeted to suppress ERK1/2 and p38 kinase MAPK in the context of OA.

### 4.3. ERK1/2 and Neoangiogenesis

New blood vessel formation, otherwise known as neoangiogenesis, constitutes a critical feature of altered synovial tissue structure that alters the activity of synovium in RA as well as OA [77]. Weng et al. [114] showed that the increase in vascular content in OA synovial tissue is associated with angiogenic stimulating proteins, such as Dickkopf-related protein-1 (Dkk-1). Wang et al. [114] also showed that IL-1β-induced Dkk-1 promoted the production of hypoxia-inducible factor-1α and additional angiogenic factors ADAMTS-5 and MMP-13 by synovial fibroblasts, as well as promoting vascular changes in endothelial cells. Moreover, stabilization of ERK, along with other proteins, reduced the effect of Dkk-1 as well as proteinase gene expression in synovial fibroblasts.

### 4.4. ERK1/2 and OA Chondrocytes

Many of the aforementioned studies were conducted on normal chondrocytes in order to probe the effect of ERK1/2 activation on chondrocyte gene expression. However, studies conducted with human chondrocytes isolated from OA cartilage also implicated ERK and mechanistic target of rapamycin (mTOR) in phospholipase-Cγ1-mediated MMP-13 gene expression [115]. Another interesting facet regarding the relationship between MMP-13 and ERK1/2 was reported by Raggatt et al. [116], who used rabbit chondrocytes to show that the internalization of MMP-13 induced the phosphorylation of ERK1/2 (p-ERK1/2), which was inhibited by receptor-related protein-1 but not by low-density lipoprotein receptor-related protein-1. The results of this study [116] suggested a mechanism involving a positive feedback loop between MMP-13 and ERK1/2-regulated ERK1/2 activation, which appears to constitute an operative mechanism between MMP-13 and p-ERK1/2.

### 4.5. ERK1/2 Signaling Pathway in Osteoblasts and Osteoclasts

ERK1/2 signaling has proven to be a critical player in regulating the functions of osteoblasts/osteocytes and osteoclasts. A study using the murine osteocyte line, MLO-Y4, [117] showed that both ERK1/2 and PI3K signaling were activated, along with essential components toward an increase in *IL-6* gene expression, when these cells were placed under cyclical compressive forces. The ERK1/2 inhibitor, PD98059, and the PI3K inhibitor, LY294002, caused *IL-6* gene expression to decrease when employed as either individual small molecule inhibitors or when these inhibitors were used together, indicating a synergistic effect of the two signaling molecules [106]. This result suggested that the ERK1/2 and PI3K pathways can play a role in promoting a pro-inflammatory state by increasing *IL-6* gene expression.

Additional studies point to the importance of ERK1/2 signaling in osteoblast differentiation and, therefore, as a contributor to osteogenesis. In this regard, Prasadam et al. [118] showed that when bone cells were cultured in the conditioned medium from OA articular chondrocytes, osteogenic gene expression was increased in subchondral bone osteoblasts as a function of ERK1/2 phosphorylation. Subchondral bone osteoblasts showed an increase in p-ERK1/2 in conjunction with increased matrix deposition and gene expression of alkaline phosphatase, osteopontin, osteocalcin, and Core Binding Factor α1 (*CBFA1*) mRNA in the presence of OA chondrocyte conditioned medium. Applying the ERK inhibitor, PD98059, decreased these indicators of osteogenesis. In a similar fashion, Choi et al. [119] measured the activity of osterix, a novel-zinc finger transcription factor in C2C12 mesenchymal cells, which could be induced by treatment with bone morphogenetic factor-2 to undergo osteoblastic differentiation. Osterix is essential for osteoblast differentiation and bone formation since it acts downstream of Runx2/CBFA1 [106]. The C2C12 cells constitutively activated MEK. Thus, ERK activation resulted in an increase in osterix transcriptional activity and the stabilization of osterix protein levels. Taken together, these studies indicated that ERK1/2 signaling played a role in the differentiation and activation of osteoblasts, resulting in an increase in bone metabolism that could then proceed to the sclerosis of bone, the latter being a defining feature of OA.

Conversely, Prasadam et al. [104] showed that culturing subchondral bone osteoblasts with the conditioned media from OA chondrocytes could also lead to increased *MMP-1* and *MMP-2* gene expression, a finding that was once again reversed by the ERK inhibitor, PD98059. Furthermore, Nakai et al. [120] showed that angiotensin II acting via the AT1 receptor led to phosphorylated ERK1/2, which induced *MMP-3* and *MMP-13* gene expression by the rat osteosarcoma cell line ROS17/2.8, a finding that was completely blocked when the ERK inhibitor, PD98059, or losartan, a selective competitive angiotensin II receptor type 1 antagonist, was added. These findings showed that 1) the ERK signaling pathway likely plays a critical role in MMP-related degradation of the bone matrix, an important component of bone remodeling during extracellular matrix turnover and bone homeostasis and 2) that Ang II activity is important for increasing the expression of MMPs, with activation of NF-κB playing a role in that response [121]. This finding may also be pertinent to aggressive bone matrix turnover as a prominent feature of RA and OA. In this regard, Shimizu et al. [122] showed that the treatment of rabbit osteoblasts with angiotensin resulted in increased expression of RANKL, a finding that was reversed by the MEK inhibitor, U0126.

The microenvironment that bone cells or their precursors are exposed to in cell culture is also likely to influence the contribution that ERK1/2 signaling makes to osteoblast differentiation. For example, Tsai et al. [123] compared the culture of mesenchymal stem cells on several different substrates including Type I collagen, fibronectin, laminin, gelatin, and poly- L-Lysine scaffolds. The results of this study showed that out of these five substrates, only Type I collagen was able to significantly upregulate osteogenic biomarkers that were accompanied by ERK1/2 activation [123]. This finding suggested that the microenvironment that osteoblasts are exposed to in vitro may play a crucial role in how ERK1/2 signaling contributes to the outcomes of experiments with bone cells or their respective progenitors.

Experimental studies in cell culture also provide evidence that ERK1/2 activation is a necessary component required for both osteoclast differentiation [124,125] and apoptosis [126]. For example, human differentiated osteoclasts undergo apoptosis when challenged with prostaglandin D2 via the chemoattractant receptor homologous molecule expressed on T-helper type 2 cell (CRTH2), which also involves β1-arrestin (Figure 1), ERK1/2, and Akt, but not inhibitor of nuclear factor kappa-B kinase subunit beta (IKK2)/NF-κB signaling [126]. In contrast, insulin was also shown to induce the expression of receptor activator of nuclear factor κB (RANK)/ receptor activator of nuclear factor κB ligand (RANKL) through ERK1/2 activation, which favored the differentiation of bone marrow macrophages to osteoclasts that were identified by tartrate-resistant acid phosphatase-positive (TRAP^+^) cells and the expression of dendritic cell-specific transmembrane protein (DC-STAMP), the d2 isoform of vacuolar (H^+^) ATPase, as well as the Vo domain genes that are involved in the fusion of osteoclastic fusion [127]. Conversely, A2B adenosine receptor stimulation was proven to be an inhibitor of activated ERK1/2, p38 MAPK, and NF-κB after RANKL/RANK stimulation, which resulted in the suppression of osteoclastic gene biomarkers and was suggested as one of the underlying mechanisms responsible for a decrease in bone resorption [128].

### 4.6. Interaction between Bone Cells and Articular Chondrocytes

The direct interaction between subchondral osteoblasts and articular chondrocytes is an area of active research, which attempts to understand the complex networking that drives destruction of cartilage in OA [129]. In this regard, Prasadam et al. [130] showed that the interaction between subchondral osteoblasts and articular chondrocytes was dependent on activation of ERK1/2 signaling. Furthermore, the de-phosphorylation of p38 MAPK also caused altered chondrocyte function to occur, reminiscent of the cartilage hypertrophic phenotype that characterizes chondrocytes in OA cartilage. Of note, constitutive expression of Spry1 (a member of the Spry family of proteins that regulate MAPK kinase activity via FGF signaling) in chondrocytes reduced FGFR2 degradation through sustained phosphorylation of ERK, the upregulation of cyclin-dependent kinase inhibitor 1 (p21Cip) and Signal Transducer and Activators of Transcription-1 (STAT1) [131]. Therefore, a further understanding of these cellular events might also be useful in explaining the disruption in the function of chondrocytes that accompanies genetic errors leading to abnormal chondrocyte terminal differentiation and its association with chondrodysplasias.

### 4.7. Other Aspects of ERK1/2 in Relation to Chondrocyte Homeostasis

Tissue inhibitor of metalloproteinases (TIMPs) are the endogenous inhibitors of MMPs [102]. However, in the chronic state of inflammation that exists in several forms of arthritis there is a skewing of the ratio of MMPs to TIMPs towards MMPs. Thus, Zhu et al. [132] showed that TIMP-3 expression induced by TGF-β1 in rat chondrocyte cultures was significantly inhibited by ERK1 knockdown, which was accompanied by reduced p-Smad3 activity. Of note, the knockdown of ERK2 had no effect on TIMP-3 and no effect either on p-Smad3 nor TGF-β1-induced TIMP-3.

Much of these data stem from in vitro analysis using chondrocytes from various species. Thus, it was noteworthy that Thiel et al. [133] showed that PD184352, another small molecule inhibitor of upstream MEK whose activation is required for ERK1/2 to be phosphorylated, inhibited paw edema and the severity of arthritis in a collagen-induced arthritis model in DBA/1LacJ mice. These results also showed a strong relationship between p-ERK and stromelysin (MMP-3), by which MMP-3 production was inhibited by PD184352 and whereby PD184352-treated mice showed less weight loss and fewer histopathologic changes in the affected arthritic joint compared to the control group.

### 4.8. Regulation of ERK1/2 by MicroRNA

In addition to the classic epigenetic regulators of ERK activation exhibited by HDAC, recently published results have also implicated microRNAs (miR) as a crucial component for the “fine-tuning” of various signaling pathways, including those of chondrocytes [134] and osteoblasts [135]. MicroRNAs are a separate category of epigenetic regulators consisting of small non-coding RNAs, generally of 20–22 nucleotides in length. In this regard, Bluhm et al. [136] showed that in chondrocytes, miR-322 targets the RAF/MEK/ERK pathway. Thus, increasing the concentration of miR-322 was shown to stabilize expression of the *Mek* gene, which led to reduced phosphorylation of ERK1/2, whereas cartilage-specific inactivation of miR-322 in mice was associated with not only decreased MEK1 levels but also with an increase in the activation of RAF/MEK/ERK. It may also be pertinent that we had shown that inhibition of MEK by the MEK1/2 inhibitor, U0126, increased the frequency of apoptotic cells in a line of immortalized human chondrocytes in response to pathologic levels of TNF-α [137]. This finding suggested that MEK as an upstream regulator of ERK1/2 likely plays a fundamental role in sustaining chondrocyte viability and cartilage repair responses. However, the relationship between the inhibition of MEK in cultured chondrocytes brought about by a pharmacologic intervention and experimentally-induced reductions in specific miR levels in vivo must be further studied. This assumption is made in light of a recent finding that linked downregulation of miR-340-5p in mice to increased chondrocyte proliferation and reduced apoptosis through inhibition of ERK signaling and fibromodulin [*FMOD*], the target gene of mIR-340-5p [138]. Fibromodulin is a regulator of the final properties of collagen matrices [139]. It is also likely that miRs play a relevant role in the accentuated bone matrix loss characteristic of osteoporosis. This provides the underpinning for identifying miRs that curtail or promote specific genes pertinent to bone formation versus pathologic bone loss as a potentially important advance in the treatment of osteoporotic bone [135].

## 5. Conclusions and Future Perspectives

It has become increasingly evident that new drug development for the treatment of various forms of cancer and arthritides will be based on our increased understanding of the role of ERK1/2 signaling, which lags far behind the therapies developed against other targets that also play a role in diseases associated with chronic inflammation. Perhaps one can argue that the increased focus on other signaling pathways besides the ERK1/2 pathway, including PI-3K/Akt/mTOR [67] and JAK/STAT [42], has occurred because they have been implicated in diseases characterized by chronic inflammation. Thus, many targets have been justified for further study based on results of experimental and pre-clinical studies, and some studies have resulted in the development of new drugs, including those targeting IL-12/IL-23/p40 (i.e., ustekinumab) [140], IL-23/p19 (i.e., guselkumab) [141], IL-17A (i.e., secukinumab; ixekizumab) [142,143], IL-17R (i.e., brodalizumab) [143], and IL-23 (i.e., tidrakizumab) [143]. There has also been a renewed interest in developing MMP, ADAMTS, and A Disintegrin and Metalloproteinase (ADAMs) inhibitors [144], since these molecules are the product of MAPK and/or JAK/STAT signaling in response to IL-1β, IL-6, IL-17 and TNF-α [144]. Furthermore, it is likely that p38γ and p38δ MAPK in response to these cytokines plays a key role in MMP gene expression in chronic inflammation [145].

However, a complication has arisen regarding the choices that must be made in any decision to further develop new drugs for specific diseases. This will entail making advances in our general understanding of how many of the pro-inflammatory cytokines activate more than one signaling pathway. This will also inform the MMPs, ADAMTS, and ADAMs that drive the progression of cell proliferation and tissue destruction, as well as also activating various transcription factors that play a role in the progression of these diseases [101]. In addition, the activation of more than one signaling pathway [146] in response to treatment of cells with a single pro-inflammatory cytokine must be considered. Such a response was noted when TNF-α was shown to activate p38 kinase (i.e., p-p38 kinase) and JNK1/2 (i.e., p-JNK1/2) as well as STAT3 (i.e., p-STAT3) in cultured human chondrocytes enzymatically dissociated from end-stage OA cartilage [147]. This finding supported the possibility that both MAPK and JAK/STAT signaling could be activated by a single cytokine through ERK1/2 as depicted in Figure 1, although based on the schematic shown in Figure 1 it may just be that TNF-α activation of ERK1/2 leads to increased *STAT3* gene expression. Obviously, further study is required to sort this all out.

Clinical inhibitors of the ERK cascade have been developed over the past decade or so [148,149,150,151,152,153,154,155,156,157]. The MEK inhibitors that are being clinically tested include trametinib, selumetinib, pimasertib, refametinib, PD-0325901, MEK162, TAK733, RO5126766, WX-554, RO4987655, cobimetinib, AZD8330, MSC2015103B, and ARRY-300. However, it also remains to be determined as to how effective certain targeted inhibitors of ERK1/2 [157] (e.g., ulixertinib, MK-8353, GDC-0994) will be, as they are presently being evaluated in various clinical trials involving patients with advanced/metastatic solid tumors. Additional anti-cancer targets have been identified that are designed to disrupt cancer cell survival. These include inhibitors of RAF [158,159], IQGAP [160], and dual inhibitors of RAF-MEK-ERK and PI3K/PDK1-AKT pathways [161].

It is also anticipated that some of these anti-MEK/ERK1/2 cascade agents could eventually be employed for evaluation in patients diagnosed with RA, PsA, as well as bone and inflammatory bowel diseases, going forward.
